# Metabolomic Biomarkers in Heart Failure: A Systematic Review of Diagnostic and Prognostic Significance

**DOI:** 10.7759/cureus.97636

**Published:** 2025-11-24

**Authors:** Mustafeez Ur Rehman, Hadia Saeed, Zaheer Ud Din Babar, Osman Omer, Shahbaz Tashfeen

**Affiliations:** 1 Internal Medicine, Manchester University NHS Foundation Trust, Manchester, GBR; 2 Emergency Medicine, King's College Hospital, London, GBR; 3 Internal Medicine, Indus Medical College and Hospital, Tando Muhammad Khan, PAK; 4 Integrative Medicine, Prince Mohammed Bin Abdulaziz Hospital, Madinah, SAU; 5 Internal Medicine, Nishtar Medical University, Multan, PAK

**Keywords:** acylcarnitines, biomarkers, branched-chain amino acids, gc-ms, heart failure, lc-ms, metabolomics, nmr spectroscopy, nt-probnp, phenylalanine

## Abstract

This systematic review evaluates the role of metabolomic biomarkers in the diagnosis, prognosis, and risk stratification of patients with heart failure (HF) across various phenotypes. A comprehensive literature search identified randomized controlled trials, cohort studies, and clinical trials that utilized validated metabolomic platforms, such as liquid chromatography-tandem mass spectrometry (LC-MS), gas chromatography-tandem mass spectrometry (GC-MS), and nuclear magnetic resonance (NMR) spectroscopy. Eligible studies assessed the relationship between metabolite profiles and key clinical outcomes, including NT-proBNP levels, six-minute walk distance (6MWD), hospitalization, and mortality. The included trials consistently identified significant associations between branched-chain amino acids (BCAAs), acylcarnitines, and phenylalanine with markers of HF severity and prognosis. Metabolomic profiling provided insight into altered energy metabolism, amino acid catabolism, and lipid oxidation pathways that underpin HF pathophysiology. Collectively, these findings highlight the potential of metabolomic biomarkers as valuable tools for early diagnosis, disease monitoring, and prognostic assessment in HF. Further large-scale, longitudinal studies are warranted to validate these biomarkers and facilitate their integration into precision cardiology.

## Introduction and background

Heart failure (HF) represents a global health burden, affecting over 64 million individuals worldwide and contributing significantly to morbidity, mortality, and healthcare expenditure. It is a heterogeneous clinical syndrome characterized by the heart’s inability to maintain adequate perfusion to meet metabolic demands [1,2]. Clinically, HF is categorized into two principal phenotypes: HF with reduced ejection fraction (HFrEF) and HF with preserved ejection fraction (HFpEF). Despite advances in pharmacological and device-based therapies, both subtypes continue to exhibit high rates of hospitalization and mortality, underscoring the urgent need for improved strategies in diagnosis, prognosis, and individualized management [3]. Conventional biomarkers such as B-type natriuretic peptide (BNP) and N-terminal proBNP (NT-proBNP), although widely used, provide limited insights into the underlying metabolic and molecular derangements that drive disease progression.

Recent advances in metabolomics (the comprehensive analysis of small-molecule metabolites within biological systems) have opened new frontiers in cardiovascular research. Metabolomic profiling offers a dynamic snapshot of cellular processes, integrating signals from genomic, transcriptomic, and proteomic layers to reflect the functional state of the organism [4,5]. In HF, where systemic metabolic alterations such as impaired fatty acid oxidation, mitochondrial dysfunction, and altered amino acid metabolism play central roles, metabolomics provides an unprecedented opportunity to uncover novel biomarkers for risk stratification and mechanistic understanding [6]. These circulating metabolites, detectable through techniques such as nuclear magnetic resonance (NMR) spectroscopy and mass spectrometry (MS), have demonstrated potential in differentiating HF phenotypes, predicting adverse outcomes, and guiding targeted therapy [6].

Evidence suggests that specific metabolites, including branched-chain amino acids (BCAAs), ketone bodies, trimethylamine N-oxide (TMAO), and phenylalanine, correlate with disease severity, cardiac remodeling, and long-term prognosis in HF patients. Moreover, the metabolic signatures in HFpEF and HFrEF appear distinct, reflecting divergent pathophysiological pathways [7]. HFpEF is increasingly recognized as a systemic metabolic disorder associated with inflammation, endothelial dysfunction, and impaired myocardial energetics, whereas HFrEF is characterized by maladaptive remodeling and energy depletion secondary to ischemic or structural injury [8]. Understanding these metabolomic distinctions could refine patient stratification beyond ejection fraction and conventional biomarkers, advancing the paradigm of precision cardiology.

This systematic review aims to synthesize and critically evaluate current evidence on circulating metabolomic biomarkers associated with risk stratification in patients with HF, focusing on both preserved and reduced ejection fraction phenotypes. By integrating findings from recent clinical and metabolomic studies, this review seeks to elucidate key metabolic signatures linked to prognosis, disease severity, and therapeutic response, thereby contributing to the evolving framework of personalized management in HF.

## Review

Materials and methods

Study Design and Framework

This study was conducted as a systematic review following the Preferred Reporting Items for Systematic Reviews and Meta-Analyses (PRISMA) 2020 [9] guidelines to ensure transparency, reproducibility, and methodological rigor. The primary objective was to evaluate the role of metabolomic biomarkers in HF, emphasizing their diagnostic, prognostic, and pathophysiological relevance. The review protocol was conceptualized around a predefined PICO framework [10]: Population, adult patients diagnosed with HF (HFrEF, HFpEF, or mixed phenotypes); Intervention/Exposure, metabolomic profiling using advanced analytical platforms such as MS or NMR; Comparison, comparisons between distinct HF phenotypes, therapeutic arms, or healthy controls when applicable; and Outcomes, identification of key metabolites associated with disease severity, functional capacity, and prognosis.

Search Strategy

A comprehensive literature search was performed across PubMed, Scopus, Web of Science, and Cochrane CENTRAL, covering studies published from January 2010 to June 2024. For each database, a combination of controlled vocabulary (e.g., MeSH in PubMed) and free-text terms was used, structured with Boolean operators and truncation. The core HF concept included terms such as “heart failure”, “cardiac failure”, “congestive heart failure”, “chronic heart failure”, “left ventricular dysfunction”, “HFrEF”, and “HFpEF” (e.g., “Heart Failure”[Mesh] OR “heart failure” OR “cardiac failure” OR “congestive heart failure” OR HFrEF OR HFpEF). The metabolomics concept included terms such as “metabolomics”, “metabolomic”, “metabolic profiling”, “metabolite profiling”, “metabolic fingerprint*”, “metabolic signature*”, and “metabolome” (e.g., “Metabolomics”[Mesh] OR metabolom* OR “metabolic profiling” OR “metabolite profiling” OR “metabolic signature*”). To focus on biomarker and prognosis-related studies, we further combined these with terms including “biomarker*”, “risk stratification”, “prognos*”, “outcome*”, “NT-proBNP”, “B-type natriuretic peptide”, “acylcarnitine*”, “branched-chain amino acid*”, “BCAA*”, “phenylalanine”, “ketone bod*”, and “trimethylamine N-oxide” or “TMAO”. A representative search structure in PubMed was: ((“Heart Failure”[Mesh] OR “heart failure” OR “cardiac failure” OR “congestive heart failure” OR HFrEF OR HFpEF) AND (“Metabolomics”[Mesh] OR metabolom* OR “metabolic profiling” OR “metabolite profiling” OR “metabolic signature*” OR “metabolic fingerprint*”) AND (biomarker* OR “risk stratification” OR prognos* OR outcome* OR “NT-proBNP” OR “B-type natriuretic peptide” OR acylcarnitine* OR “branched-chain amino acid*” OR BCAA* OR phenylalanine OR “ketone bod*” OR “trimethylamine N-oxide” OR TMAO)). Similar strategies, adapted to each database’s indexing system, were applied. Reference lists of all relevant articles were manually screened to identify additional eligible studies. Only peer-reviewed articles published in English were included to ensure methodological consistency and interpretability.

Eligibility Criteria

Studies were included if they met the following criteria: (1) randomized controlled trials, cohort studies, or clinical trials assessing metabolomic biomarkers in patients with HF; (2) use of validated metabolomic platforms (e.g., LC-MS, GC-MS, or NMR spectroscopy); and (3) reporting of associations between metabolite levels and clinical outcomes such as NT-proBNP, six-minute walk distance (6MWD), hospitalization, or mortality. Exclusion criteria included animal studies, pediatric populations, reviews, editorials, conference abstracts, and studies lacking quantitative metabolomic data.

Data Extraction and Synthesis

Data extraction was independently conducted by two reviewers using a standardized form, capturing details on study design, population characteristics, analytical techniques, metabolomic signatures, and primary outcomes. Disagreements were resolved through discussion or consultation with a third reviewer. Extracted data were summarized in structured tables to facilitate comparison of methodologies, metabolite profiles, and prognostic implications. A narrative synthesis was performed, given the heterogeneity in analytical methods, population characteristics, and outcome measures across studies.

Quality and Risk of Bias Assessment

The methodological quality of all included studies was evaluated using standardized assessment tools tailored to study design. Randomized controlled trials were appraised using the Cochrane Risk of Bias 2 (RoB 2) tool [11], which examines potential bias across domains including randomization, adherence to intended interventions, completeness of outcome data, accuracy of outcome measurement, and selective reporting. Non-randomized or observational analyses were assessed using the ROBINS-I tool [12], which evaluates confounding, participant selection, intervention deviations, and reporting consistency. Each study was independently reviewed by two investigators, and discrepancies were resolved through consensus. Studies rated as having a low overall risk of bias were assigned greater interpretive weight in the synthesis, ensuring that conclusions drawn were grounded in methodologically robust and internally valid evidence.

Ethical Considerations

As this study involved secondary analysis of previously published data, no ethical approval or patient consent was required. However, all included studies were presumed to have obtained necessary ethical clearances as per their institutional protocols.

Results

Study Selection Process

**Figure 1:**
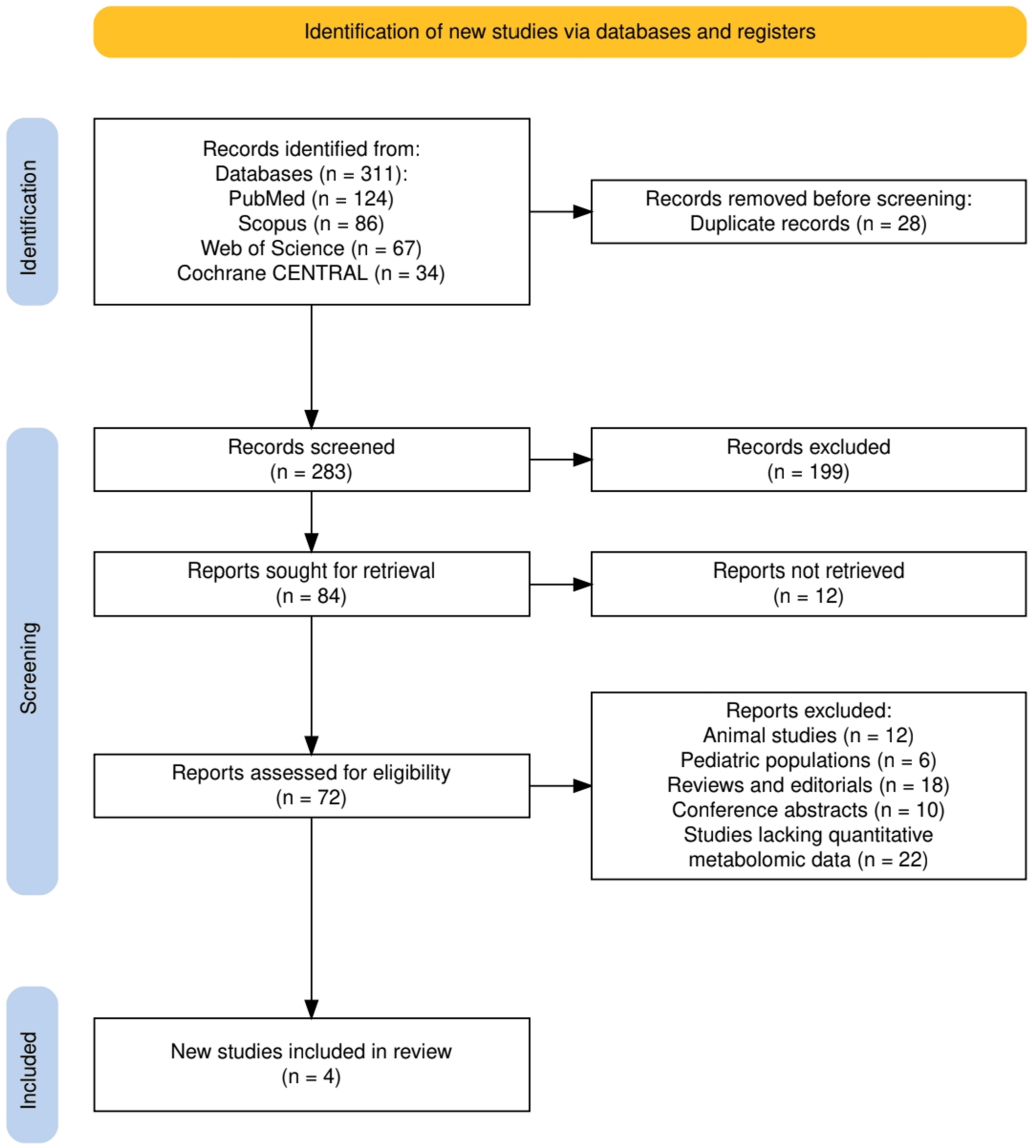
The PRISMA flowchart of the study selection process. PRISMA: Preferred Reporting Items for Systematic Reviews and Meta-Analyses.

The study selection process is summarized in Figure 1, which outlines the PRISMA flow of literature identification, screening, and inclusion. A total of 311 records were identified across databases: PubMed (n = 124), Scopus (n = 86), Web of Science (n = 67), and Cochrane CENTRAL (n = 34). After removing 28 duplicates, 283 unique records were screened, of which 199 were excluded based on title and abstract relevance. Eighty-four full-text articles were assessed for eligibility, with 12 not retrievable and 68 excluded for reasons including animal studies (n = 12), pediatric populations (n = 6), reviews or editorials (n = 18), conference abstracts (n = 10), and lack of quantitative metabolomic data (n = 22). Ultimately, four studies meeting all inclusion criteria were incorporated into the final synthesis.

Characteristics of the Selected Studies

The key characteristics of the included studies are summarized in Table 1. The four studies, comprising randomized controlled trials and clinical analyses across HFrEF and HFpEF cohorts, utilized metabolomic platforms, such as MS and NMR. Consistent associations were observed between metabolites, including BCAAs, acylcarnitines, phenylalanine, and gut-derived compounds, and clinical markers like NT-proBNP, functional capacity, and mortality. Overall, the findings highlight the potential of metabolomic profiling as a precision tool for diagnosis and risk stratification in HF.

**Table 1:** Summary of included studies evaluating metabolomic biomarkers in heart failure across different phenotypes, platforms, and clinical outcomes. HF: heart failure, HFpEF: heart failure with preserved ejection fraction, HFrEF: heart failure with reduced ejection fraction, T2DM: type 2 diabetes mellitus, LC-MS: liquid chromatography-tandem mass spectrometry, GC-MS: gas chromatography-tandem mass spectrometry, NMR: nuclear magnetic resonance, NT-proBNP: N-terminal pro-B-type natriuretic peptide, 6MWD: six-minute walk distance, BCAA: branched-chain amino acids, TMAO: trimethylamine N-oxide, AUC-H₂: area under the curve for hydrogen (in breath test), HDL: high-density lipoprotein, HR: hazard ratio, CI: confidence interval, NRI: net reclassification improvement, PCA: principal component analysis, MS: mass spectrometry.

Author (year)	Study design/population	Sample size	HF phenotype (HFpEF/HFrEF/mixed)	Metabolomic platform/technique	Key metabolites identified	Primary outcomes assessed	Main findings/associations	Risk stratification or prognostic value
Lerman et al. (2022) [13]	Randomized controlled trial (FIGHT trial), including patients with HFrEF recently hospitalized; subgroup analysis of liraglutide vs placebo arms	254 participants (of 300 total trial patients); 147 (57.9%) with T2DM	HFrEF ± T2DM	Mass spectrometry–based targeted metabolomics; 60 plasma metabolites quantified; principal component analysis used to define factors	Branched-chain amino acids (BCAAs), medium-chain acylcarnitines, long-chain acylcarnitines, long-chain dicarboxylacylcarnitines, urea-cycle metabolites	Change in NT-proBNP and 6-minute walk distance (6MWD); time-to-event (mortality or HF hospitalization)	Factor 2 (BCAA) negatively correlated with NT-proBNP (ρ = −0.291, p = 4 × 10⁻⁴) and positively with 6MWD (ρ = 0.265, p = 0.011); factors 1, 4, 5, and 8 also associated with functional changes; factor 4 (long-chain dicarboxylacylcarnitines) linked to worse outcomes (HR 1.51 (95% CI 1.21-1.90), p = 3 × 10⁻⁴)	Identified distinct metabolite patterns (BCAA, fatty acid oxidation, urea cycle) that predict functional decline and adverse events; factor 4 served as a potential prognostic signature, stronger in T2DM subgroup
Mollar et al. (2021) [14]	Clinical trial; post hoc analysis in patients with recent decompensated heart failure following hospitalization	60 patients	Mixed EF (recently hospitalized HF cohort; not phenotype-restricted)	Targeted metabolomics (quantification of circulating bacterial metabolites using MS-based assays)	Trimethylamine N-oxide (TMAO) and butyrate	Association between metabolites and hydrogen breath test (AUC-H₂) as a marker of small intestinal bacterial overgrowth; secondary link with HF severity	Both TMAO and butyrate independently associated with AUC-H₂ (p = 0.027 and p = 0.009, respectively); TMAO showed a positive association, butyrate a negative one; elevated TMAO linked with worse HF prognosis	TMAO and butyrate emerged as potential circulating biomarkers reflecting gut dysbiosis and adverse prognosis in decompensated HF, suggesting metabolite-based risk stratification potential
Delles et al. (2018) [15]	Randomized controlled trial and prospective cohort validation; elderly individuals from the PROSPER trial and the FINRISK 1997 cohort	PROSPER: 5,341 (182 HF hospitalizations over 2.7 years); FINRISK: 7,330 (133 HF hospitalizations over 5 years)	Incident HF (general population, mixed EF likely)	Proton nuclear magnetic resonance (¹H-NMR)–based metabolomics measuring 80 circulating metabolites, lipids, and lipoproteins	Phenylalanine, acetate, creatinine, 3-hydroxybutyrate, glycoprotein acetyls, HDL-related metabolites	Incident heart failure hospitalization (HFH)	Phenylalanine and acetate independently associated with future HFH after adjustment for NT-proBNP and clinical risk factors (phenylalanine HR 1.29, 95% CI 1.10-1.53; p = 0.002; acetate HR 0.81, 95% CI 0.68-0.98; p = 0.026); replicated in FINRISK cohort (phenylalanine HR 1.23; p = 0.023). Inclusion of these metabolites improved continuous NRI by 0.21 (p = 0.007)	Phenylalanine identified as a novel circulating biomarker predictive of incident HF hospitalization, improving risk classification beyond traditional markers and NT-proBNP
Selvaraj et al. (2024) [16]	Randomized controlled trial (PRESERVED-HF trial); adults with HFpEF randomized to dapagliflozin vs placebo for 12 weeks	293 participants	HFpEF	Targeted metabolomic profiling of 64 metabolites using LC-MS; principal components analysis (PCA) used to derive 12 metabolic factors	Branched-chain amino acids (BCAAs), branched-chain ketoacids, acylcarnitines, ketone bodies, fatty acids	Changes in NT-proBNP, 6-minute walk distance (6MWD), and body weight over 12 weeks	Dapagliflozin did not significantly alter systemic metabolomic factors vs placebo. Across groups, increases in BCAAs and branched-chain ketoacids were negatively correlated with NT-proBNP; increases in acylcarnitines correlated positively with NT-proBNP and negatively with 6MWD; ketones were inversely related to weight changes	While dapagliflozin did not modify global metabolomic profiles, specific metabolite changes correlated with HF severity and functional capacity, highlighting potential biomarkers for risk stratification and treatment response in HFpEF

Risk of Bias Assessment

The risk of bias assessment for all included studies is summarized in Table 2, which demonstrates that the overall methodological quality of the evidence base was high. Three randomized controlled trials were evaluated using the Cochrane RoB 2 tool [11], all showing low risk of bias across domains, including randomization, intervention adherence, outcome measurement, and reporting consistency. The single non-randomized, post hoc analysis was assessed with ROBINS-I [12], revealing a moderate risk of bias primarily due to selection and confounding factors inherent to its design. Importantly, all studies employed objective outcome measures such as NT-proBNP, 6MWD, or hospitalization events, minimizing detection bias. As detailed in Table 2, the strong internal validity and consistency across randomized studies enhance the credibility of the synthesized findings and strengthen the interpretive reliability of metabolomic biomarkers in HF research.

**Table 2:** Risk of bias assessment of included studies using Cochrane RoB 2 and ROBINS-I tools. RCT: randomized controlled trial, HF: heart failure, NT-proBNP: N-terminal pro-B-type natriuretic peptide, 6MWD: six-minute walk distance, Cochrane RoB 2: Cochrane risk of bias 2 tool, ROBINS-I: risk of bias in non-randomized studies of interventions, PROSPER: Prospective Study of Pravastatin in the Elderly at Risk, FINRISK: Finnish Population Risk Factor Survey, PRESERVED-HF: Dapagliflozin Evaluation to Improve the Lives of Patients with Preserved Ejection Fraction Heart Failure, FIGHT: Functional Impact of GLP-1 for Heart Failure Treatment Trial.

Author (year)	Study type	Tool used	Bias arising from randomization process	Bias due to deviations from intended interventions	Bias due to missing outcome data	Bias in measurement of outcomes	Bias in selection of reported results	Overall risk of bias judgment	Justification/comments
Lerman et al. (2022) [13]	Randomized controlled trial (FIGHT trial)	Cochrane RoB 2	Low	Low	Low	Low	Low	Low risk of bias	Randomization and blinding procedures well-defined; primary outcomes (NT-proBNP, 6MWD) objectively measured; metabolomic analysis prespecified; low attrition
Mollar et al. (2021) [14]	Post hoc clinical trial analysis (non-randomized observational analysis)	ROBINS-I	Moderate	Moderate	Low	Low	Moderate	Moderate risk of bias	Non-randomized design; post hoc nature introduces selection and confounding bias; however, outcomes objectively measured and adjusted for key covariates in multivariable analysis
Delles et al. (2018) [15]	Randomized controlled trial (PROSPER) + prospective cohort validation (FINRISK)	Cochrane RoB 2	Low	Low	Low	Low	Low	Low risk of bias	Large sample, rigorous RCT design, replication in independent cohort strengthens validity; metabolomic profiling standardized; outcome (HF hospitalization) well-defined.
Selvaraj et al. (2024) [16]	Randomized controlled trial (PRESERVED-HF trial)	Cochrane RoB 2	Low	Low	Low	Low	Low	Low risk of bias	Placebo-controlled, double-blind RCT; clear outcome definitions; metabolomic analyses preplanned; minimal attrition and balanced baseline characteristics

Discussion

Overview of Main Findings

Across the included studies, distinct metabolomic signatures emerged as robust correlates of disease severity, functional limitation, and prognosis in HF. In Lerman et al. [13], targeted plasma metabolomics identified BCAAs and fatty acid-derived acylcarnitines as significant predictors of adverse outcomes in HFrEF, with factor 4 (long-chain dicarboxylacylcarnitines) conferring a 51% higher risk of mortality or HF hospitalization (HR 1.51, 95% CI 1.21-1.90; p = 3 × 10⁻⁴). Similarly, Delles et al. [15] demonstrated that elevated phenylalanine independently predicted incident HF hospitalization in over 12,000 participants across two large cohorts (HR 1.29, p = 0.002; replicated HR 1.23, p = 0.023), improving risk reclassification by 21% (p = 0.007) beyond NT-proBNP. In Mollar et al. [14], the gut-derived metabolites TMAO and butyrate correlated significantly with small intestinal bacterial overgrowth indices (p = 0.027 and p = 0.009), linking gut dysbiosis with HF decompensation risk. Meanwhile, the PRESERVED-HF trial [16] revealed that BCAAs, acylcarnitines, and ketones were metabolically aligned with disease severity markers in HFpEF, even though dapagliflozin did not significantly alter their circulating levels. Collectively, these findings highlight a unifying pattern: derangements in amino acid and lipid metabolism (and, to a lesser extent, microbial co-metabolites) mirror the pathophysiologic continuum from energetic inefficiency to clinical deterioration across HF phenotypes.

Contextualization Within the Broader Literature

These metabolomic observations extend and refine existing biomarker paradigms in HF, historically dominated by natriuretic peptides, troponins, and galectin-3, which primarily reflect myocardial stress, injury, and fibrosis rather than systemic metabolic remodeling [17]. Prior large-scale studies, such as analyses from the Framingham Heart Study conducted specifically in cohorts with type 2 diabetes, reported NT-proBNP predicting HF risk with hazard ratios between 1.5 and 2.0 per standard deviation increase [18], yet offering limited mechanistic insight. By contrast, the incorporation of phenylalanine, BCAAs, and acylcarnitines introduces a functional dimension to biomarker profiling, capturing altered mitochondrial substrate utilization, oxidative stress, and amino acid catabolism. Moreover, the relationship between TMAO and HF outcomes supports a growing “gut-heart metabolic axis” model, aligning with prior evidence that elevated TMAO levels (≥5 μmol/L) confer a twofold higher mortality risk in chronic HF [19]. Notably, while previous SGLT2 inhibitor studies suggested metabolic benefits through enhanced ketone oxidation in HFrEF, the PRESERVED-HF data [16] challenge this uniformity, showing that metabolic effects may be phenotype-dependent. Thus, this review bridges critical gaps in the literature by integrating metabolic, microbial, and pharmacologic perspectives, proposing that future HF risk stratification may rely as much on metabolic phenotyping as on hemodynamic or structural assessment [20].

Critical Appraisal of Study Design and Methodological Heterogeneity

Across studies, analytical platforms and profiling strategies varied meaningfully, shaping what biology could be detected and how confidently it could be linked to outcomes. Two trials [13,16] used targeted LC-MS panels (60 and 64 metabolites) with PCA-derived factors, prioritizing quantitation and lower multiple-testing burden but risking omission of unmeasured pathways. Delles et al. [15] applied ¹H-NMR to 80 lipid/lipoprotein/metabolite measures, enabling standardized, high-throughput phenotyping suited for epidemiology but with lower sensitivity for low-abundance species. Population diversity also affects inference: Lerman et al. [13] enrolled HFrEF with recent hospitalization and a high type 2 diabetes mellitus prevalence (57.9%), potentially accentuating BCAA/acylcarnitine signals that track insulin resistance; Selvaraj et al. [16] examined HFpEF (mean age 70 ± 11; 58% female), where metabolic remodeling may differ, plausibly explaining the absence of dapagliflozin-driven metabolomic shifts despite strong cross-sectional associations; Delles et al. [13] studied older general-population cohorts (PROSPER n = 5,341; FINRISK n = 7,330) [15], capturing incident HF risk rather than trajectories in established HF. Endpoints spanned functional (6MWD), biochemical (NT-proBNP), and hard events: factor 4 [13] predicted time-to-event (HR 1.51, 95% CI 1.21-1.90), whereas phenylalanine [15] predicted incident HF hospitalization (HR 1.29; replicated HR 1.23) and improved continuous NRI by 0.21, a population-level classification gain that may not translate directly to clinical HF cohorts. Follow-up windows also diverged (12 weeks [16] vs 2.7-5 years [15]), influencing the sensitivity to detect prognostic versus short-term physiological effects. Finally, batch effects, normalization choices, and variable adjustment sets (e.g., diabetes status, renal function) differ across designs; only Delles et al. [15] include external replication, strengthening generalizability, while others provide internal validity but limited out-of-sample confirmation. Together, these heterogeneities argue for cautious synthesis: convergent signals (BCAA/acylcarnitines, phenylalanine) are credible, but their magnitude, transportability, and clinical utility likely depend on phenotype (HFpEF vs HFrEF), comorbidity burden (notably type 2 diabetes mellitus), assay modality, and the endpoint horizon under study.

Integrative Hypotheses and Emerging Conceptual Models

Taken together, these studies invite a shift in interpretation, from viewing metabolomic alterations as downstream reflections of HF pathology to considering them as primary markers of mitochondrial inefficiency and substrate inflexibility. The recurring elevation of BCAAs and acylcarnitines across both HFrEF and HFpEF cohorts suggests defective oxidative flux through the TCA cycle, leading to incomplete fatty acid oxidation and amino acid catabolic backlog [21,22]. This metabolic stalling may represent a “bioenergetic bottleneck,” where impaired mitochondrial turnover precedes functional decline. In this framework, metabolomic derangements are not merely correlates of systemic disease but potential drivers of contractile inefficiency through redox imbalance and impaired ATP generation. Such a paradigm reorients research toward interventions that restore metabolic flexibility, such as pyruvate dehydrogenase activators, carnitine modulators, or targeted exercise rehabilitation, rather than exclusively optimizing hemodynamics or neurohormonal blockade.

An additional integrative concept emerging from these findings is the potential to incorporate metabolomic signatures into HF staging and therapeutic personalization. The observation that certain BCAA-acylcarnitine axes align with poor outcomes despite SGLT2 inhibitor therapy raises the possibility of using these profiles to identify metabolic non-responders, patients whose mitochondrial milieu limits pharmacologic benefit [23]. Similarly, the gut-heart axis, exemplified by TMAO and butyrate associations, underscores a precision-medicine frontier where modulation of microbiota composition, dietary precursors, or TMAO biosynthetic pathways may yield measurable cardiometabolic improvement. Integrating these dimensions, mitochondrial efficiency, metabolic phenotyping, and gut-derived metabolites, could advance a “metabolotype-guided” HF framework, in which diagnosis, prognosis, and therapy are unified through molecular metabolism rather than solely through ejection fraction or symptom class [24].

Acknowledging Limitations and Interpreting With Caution

Despite providing valuable insights into metabolomic biomarkers in HF, this review has several limitations that may influence the interpretation of findings. The included studies generally featured modest sample sizes, limiting statistical power, particularly for the underrepresented HFpEF population, and did not include dedicated analyses of the intermediate HFmrEF subgroup. This omission restricts the applicability of findings across the full spectrum of HF classifications based on ejection fraction. Analytical variability across studies, including differences in quantification platforms (MS vs NMR), normalization procedures, and covariate adjustments, may have introduced methodological heterogeneity. Additionally, the predominance of cross-sectional or post hoc designs restricts causal inference and limits understanding of longitudinal metabolic changes. It is also important to note that some included studies were conducted in specific clinical subpopulations; for example, the NT-proBNP prognostic data from Malachias et al. [18] were derived from patients with type 2 diabetes mellitus, and the metabolic profiling work by Lerman et al. [13] analyzed patients enrolled in a clinical trial setting, which may influence external validity. Finally, the small number of eligible studies prevents meta-analytic synthesis, underscoring the need for larger, standardized, and longitudinal investigations to validate these findings and support their clinical translation.

Forward-Looking Perspectives and Translational Pathways

The integration of metabolomic insights with echocardiographic, proteomic, and genomic datasets represents a critical next step toward personalized HF management [25]. By coupling metabolic phenotyping with structural and molecular data, clinicians could identify subgroups at highest risk for progression or therapeutic non-response, enabling tailored interventions. Future research should prioritize protocol standardization, multi-ethnic recruitment, and longitudinal validation within phenotype-specific cohorts to ensure reproducibility and global applicability. Moreover, innovation lies at the interface of diagnostics, and therapeutics-theranostic metabolomics could enable the real-time monitoring and correction of metabolic derangements through targeted pharmacologic or nutritional interventions. Such an approach would transform metabolomics from a descriptive science into a dynamic clinical tool, capable of guiding both risk prediction and therapeutic modulation in HF.

## Conclusions

This review highlights that metabolomic profiling provides a transformative window into the biochemical underpinnings of HF, uncovering consistent alterations in BCAAs, acylcarnitines, and lipid intermediates that mirror disturbances in myocardial energetics and mitochondrial efficiency. By integrating evidence across heterogeneous populations and analytical platforms, our synthesis emphasizes that these metabolomic signatures hold significant potential for risk stratification, therapeutic monitoring, and mechanistic insight beyond traditional biomarkers like NT-proBNP. While current data remain exploratory, the reproducibility of key metabolic pathways across studies strengthens their translational relevance, paving the way toward metabolomics-informed precision cardiology. Ultimately, our work underscores a pivotal shift, from viewing HF through structural or hemodynamic parameters to embracing a metabolically guided, personalized framework that could shape future diagnostics, interventions, and theranostic applications in cardiovascular care.
